# GATA-type transcription factors play a vital role in radiation sensitivity of *Cryptococcus neoformans* by regulating the gene expression of specific amino acid permeases

**DOI:** 10.1038/s41598-019-42778-7

**Published:** 2019-04-23

**Authors:** Wanchang Cui, XiangHong Li, Lisa Hull, Mang Xiao

**Affiliations:** Radiation Countermeasures Program, Armed Forces Radiobiology Research Institute, Uniformed Services University of the Health Sciences, Bethesda, MD USA

**Keywords:** Fungal biology, Transcription

## Abstract

*Cryptococcus neoformans* is a basidiomycete fungus that is highly resistant to ionizing radiation and has been identified in highly radioactive environments. Transcription factors (TFs) are master regulators of gene expression by binding to specific DNA sequences within promoters of target genes. A library of 322 signature-tagged gene deletion strains for 155 *C*. *neoformans* TF genes has been established. Previous phenome-based functional analysis of the *C*. *neoformans* TF mutant library identified key TFs important for various phenotypes, such as growth, differentiation, virulence-factor production, and stress responses. Here, utilizing the established TF mutant library, we identified 5 TFs that are important for radiation sensitivity, including *SRE1*, *BZP*2, *GAT*5, *GAT6*, and *HCM1*. Interestingly, *BZP2*, *GAT5* and *GAT6* all belong to the GATA-type transcription factors. These factors regulate transcription of nitrogen catabolite repression (NCR) sensitive genes when preferred nitrogen sources are absent or limiting. In addition to radiation, we found that specific GATA factors are important for other stressors such as rapamycin, fluconazole, and hydroxyurea treatment. Using real-time PCR method, we studied the expression of GATA down-stream genes after radiation exposure and identified that *AAP4*, *AAP5* and *URO1* were differentially expressed in the *GAT5* and *GAT6* mutants compared to the wild type cells. In summary, our data suggest that GATA TFs are important for radiation sensitivity in *C*. *neoformans* by regulating specific downstream *AAP* genes.

## Introduction

It is well established that ionizing radiation (IR) causes deleterious effects on cell survival by causing damages to cellular macromolecules including nucleic acids, proteins and lipids. However, the cellular sensitivity to IR varies greatly between different cell types. For example, mammalian cells have a D_10_ value (D_10_ is the radiation dose which decreased cell survival to 10%) about 4 Gy; most bacteria have a D_10_ value less than 200 Gy; interestingly some organisms in all three domains of life can withstand IR doses above several thousand Gy or even 12,000 Gy^1,2^. *Cryptococcus neoformans* is a basidiomycetous fungal pathogen that causes cryptococcal meningitis and responsible for 181,100 annual deaths globally^3^. Interestingly, *C*. *neoformans* was found in highly radioactive environments such as the cooling pools of nuclear reactors, the stratosphere, and the damaged nuclear reactor at Chernobyl Nuclear Power Plant^4,5^. The *C*. *neoformans* var. *grubii* (H99) strain is the most common reference strain used in the *C*. *neoformans* research community and it has a D_10_ value about 2000 Gy^5^. Many studies have been performed to understand the mechanisms of radiation resistance in *C*. *neoformans*. For example, melanin has been suggested to protect *C*. *neoformans* against IR through scavenging of free radicals generated by IR, dissipating radiation energy in the melanin polymer and converting radiation energy to metabolically useful reducing power^6,7^. One transcription factor, named Bdr1, has been suggested as a regulator of radiation resistance in *C*. *neoformans* by controlling the expression of DNA damage repair genes^5^. However, more work is still needed to understand the mechanism of radiation resistance in *C*. *neoformans*.

Transcription factors (TFs) are key regulators for gene expression coordination and their study is very important for understanding the molecular mechanisms of development and responses to environmental signals^8^. Many TFs, such as p53, nuclear factor κB (NF-κB), activated protein 1 (AP-1), nuclear erythroid-derived 2-related factor 2 (Nrf2), and cAMP responsive element binding protein (CREB) play important roles in cellular radiation responses^9^. Compared to four diverse basidiomycetes, *C*. *neoformans* are highly enriched in fungal-specific transcription factor domains^10^, which may help explain the radiation resistance of *C*.*neoformans*. Therefore, studying the TFs in the *C*. *neoformans* may provide important insights into their radiation resistance. Recently, Jung *et al*. constructed a systematic *C*. *neoformans* TF mutant library, which consists of 322 signature-tagged gene-deletion strains for 155 putative TF genes predicted by bioinformatics analysis^11^. Using this library, TFs important for 32 distinct growth conditions have been identified^11^.

In the current study, we exposed the *C*. *neoformans* TF mutant library to high dose γ-radiation, and identified several TFs that are important for radiation sensitivity. Our data showed that specific GATA family TFs are important for stress responses in *C*. *neoformans* including radiation, rapamycin, hydroxyurea and fluconazole. We also identified important amino acid permease genes that are downstream target genes of GATA TFs important for IR. In summary, our study provides better understanding of the transcriptional circuits in the *C*. *neoformans* after radiation exposure.

## Materials and Methods

### Strain and media

The Jung’s *C*. *neoformans* TF mutant library^11^ was procured from the Fungal Genetics Stock Center (FGSC, Kansas State University, Manhattan, KS). The *C*. *neoformans* H99S^10^ was a gift from Dr. Joe Heitman’s lab at Duke University Medical Center (Durham, North Carolina), which was the parent strain of the Jung’s TF mutant library. These strains were routinely grown at 30 °C in yeast extract peptone dextrose (YPD) medium. For other stress response tests, rapamycin, hydroxyurea or fluconazole was added in the YPD agar plates.

### Irradiation with γ-radiation

For survival assays, overnight cultured fungal cells were 10-fold serially diluted in PBS and 3 µL of 10^2^-10^5^ dilutions were spotted onto YPD plates. The plates were irradiated in a ^60^Co irradiator (Model 109; J. L. Shepard and Associates, San Fernando, California) at 130 Gy/min. Cells were allowed to grow for 5 days before being photographed.

For molecular biology assays, overnight cultured fungal cells were washed in PBS and then irradiated in tubes on ice in the same irradiator. The irradiated cells were either harvested immediately or diluted in 50× volume of fresh YPD medium and harvested at selected time points after incubation with shaking.

### RNA extraction and quantitative real-time PCR

RNA was extracted using the mirVana miRNA Isolation Kit (Thermo Fisher Scientific, Grand Island, New York) according to the manufacturer’s instructions. RNA concentration and purity were determined using a Nanodrop 1000 spectrophotometer (NanoDrop Technologies, Inc., Wilmington, DE). Reverse transcription (RT) was performed using the High-Capacity cDNA Reverse Transcription Kit. The resulting cDNA was quantitatively amplified using PowerUp SYBR^®^ Green Master Mix with specific primers on a 7900HT Fast Real-Time PCR System (Thermo Fisher Scientific, Grand Island, New York). The primer sequences were either from literature or self-designed as shown in Table 1. The relative mRNA expression fold change was calculated using the 2^−ΔΔT^ method with actin as the endogenous control. Each gene’s relative expression was normalized to its gene level of the H99S 1 kGy samples at 0 h.

**Table 1:** Primer sequences for qRT-PCR.

Primer	Sequence	target	References
B679	CGCCCTTGCTCCTTCTTCTATG	actin	^5^
B680	GACTCGTCGTATTCGCTCTTCG	actin	^5^
GAT1F1	GTTCGCCAACGAGTGAAAAT	GAT1	^35^
GAT1R1	GGGAAGGGTTGCTTTCTTTC	GAT1	^35^
GAT5F1	GTGAGGTGGCGGAATACCAA	GAT5	self designed
GAT5R1	GAGGGTTGTGGGTTTACCAGAT	GAT5	self designed
GAT6F2	GCCTGCCACAGCCTAATACA	GAT6	self designed
GAT6R2	CCCGCAATCATCGCAAACTT	GAT6	self designed
PRCP185	GCCTTATGGTATACTCTATGATG	AAP1	^17^
PRCP186	CCGTATGCCCGAGCACCGAGG	AAP1	^17^
PRCP187	CATGGTATACGCGATGATGG	AAP2	^17^
PRCP188	TCTGGCTCCCCAGAAGTTAATG	AAP2	^17^
AAP3F1	GCTGGTGAAGCCAAGAAT	AAP3	self designed
PRCP190	ATGTACCACCGAGATAAAAG	AAP3	^17^
PRCP191	CGAGGCAAAGAACCCACG	AAP4	^17^
PRCP192	AATCAAGATGCAAGCGTTTATG	AAP4	^17^
PRCP193	ACTTACTTGGACCTCTATCCTC	AAP5	^17^
PRCP194	TTTTCGGATCAGCTTGAAACC	AAP5	^17^
PRCP195	CCTTGAAAGACCGTTTCGGC	AAP6	^17^
PRCP196	TGTCACAAGTGTTGGGTCATTG	AAP6	^17^
AAP7F1	GGATGTTTGGCCTATCTCTC	AAP7	self designed
PRCP198	CATGTATGTGAAAGCGATGG	AAP7	^17^
PRCP199	TCTCTTTCTAGGGATTCTTATC	AAP8	^17^
PRCP200	CTCCGCCATATGGGCAGAAGC	AAP8	^17^
PRCP215	AGACCGAGGAGGAGGACGCTG	TPR2	^17^
PRCP216	AAGCGTCGAATCTAGATTGGTC	TRP2	^17^
PRCP217	AGGAGATCGAGGGAATGTTCG	TRP3	^17^
PRCP218	CGTAAGCGATCTCTCCACCG	TRP3	^17^
PRCP219	GCCCAGGCCGGTGCTTTTC	TRP4	^17^
PRCP221	ATGTATCCCATCCATCGCCAC	TRP4	^17^
PRCP222	CTTGTTCATGGGCTACTACAAC	TRP5	^17^
PRCP223	ACGCCCATCTTGGAGACGAC	TRP5	^17^
UQ1070	AAAAGCTAAAGAGAACGACGTTGC	PUT1	^20^
UQ1071	TCTCACTCTTTGACTTTGGAGGTTT	PUT1	^20^
UQ1072	AGAAGGCTAAGGAGAACAACATCATT	PUT5	^20^
UQ1073	GTCCAGATCTCCTCCTTGGAAG	PUT5	^20^
UQ1074	TCATTAACGGTGAGGAGGTCAAG	PUT2	^20^
UQ1075	GCAAGAGCACCGTCAATGG	PUT2	^20^
UQ613	TGAGTACACCCTCCGAGTCCTT	URO1	^20^
UQ733	TTTACAGTGTCGGTAGCAACGAC	URO1	^20^
UQ609	CTTATCGACACCCACGTCCA	DAL1	^20^
UQ736	GCATGTCGATCAGGGTGGT	DAL1	^20^
UQ739	TAAGATCAAACTCGCTGACATGG	URE1	^20^
UQ740	CTCGAATGACCTTACCTCCACC	URE1	^20^
UQ1586	GCTTCCCAGAATGAGCTTAACG	GDH1	^20^
UQ1587	CGTCAAGTGTACAGCCCATGTT	GDH1	^20^
UQ1588	CTTATGCTTATGTCGGATGGATCTT	GDH2	^20^
UQ1589	TGGACTCATCCAGAGCATCCT	GDH2	^20^
UQ1590	TCGAATCCCCAGGCATGT	GLN1	^20^
UQ1591	CGACGAGGATGGCTGTGACT	GLN1	^20^
UQ1592	CCGTTGGATCAGAGAAATTCTACC	GLT1	^20^
UQ1593	CTGCTGAGGACCGAGGAAAC	GLT1	^20^
UQ1630	TTATGTTTTGCTGGACTACCTTGGT	AMT1	^20^
UQ1631	CGCCACCTGCATAGTCCAAT	AMT1	^20^
UQ1632	TGAGGTTAAGGGCTAGCGAAGA	AMT2	^20^
UQ1633	CGGGATCTGTGCCAACATAGT	AMT2	^20^
URO2F	CTACCCAGTGGTTGAGATTACC	URO2	self designed
URO2R	CCACGGTATGTGGTGTAAGAA	URO2	self designed
URO3-2F	GGACATCCCATGATTGGCGA	URO3	self designed
URO3-2R	TGGTTGAGATGAGCAAGTCTCT	URO3	self designed
DAL233-2F	CCGTTGGCGGAGATCTCAAT	DAL233	self designed
DAL233-2R	GATTGAGCGGAGGGGTATGG	DAL233	self designed

### Phylogenetic tree analysis

Phylogenetic tree analysis was performed online using the protein sequence of each GATA factor with default settings at https://www.ebi.ac.uk/Tools/phylogeny/simple_phylogeny/^12^.

### Statistical analysis

Two-way ANOVA with multiple comparisons tests was performed to determine the gene expression levels compared to the wild type H99S 1 kGy group. The relative expression levels were log2 transformed. Differential gene expression was pseudocolored using the heatmap feature in GraphPad Prism 7.03 (GraphPad Software, San Diego, CA). Statistical analysis was performed using GraphPad Prism 7.03 software (GraphPad Software, San Diego, CA). All values of p < 0.05 were considered as significant differences.

## Results

### Important TFs for radiation sensitivity identified using the mutant TF library

**Figure 1:**
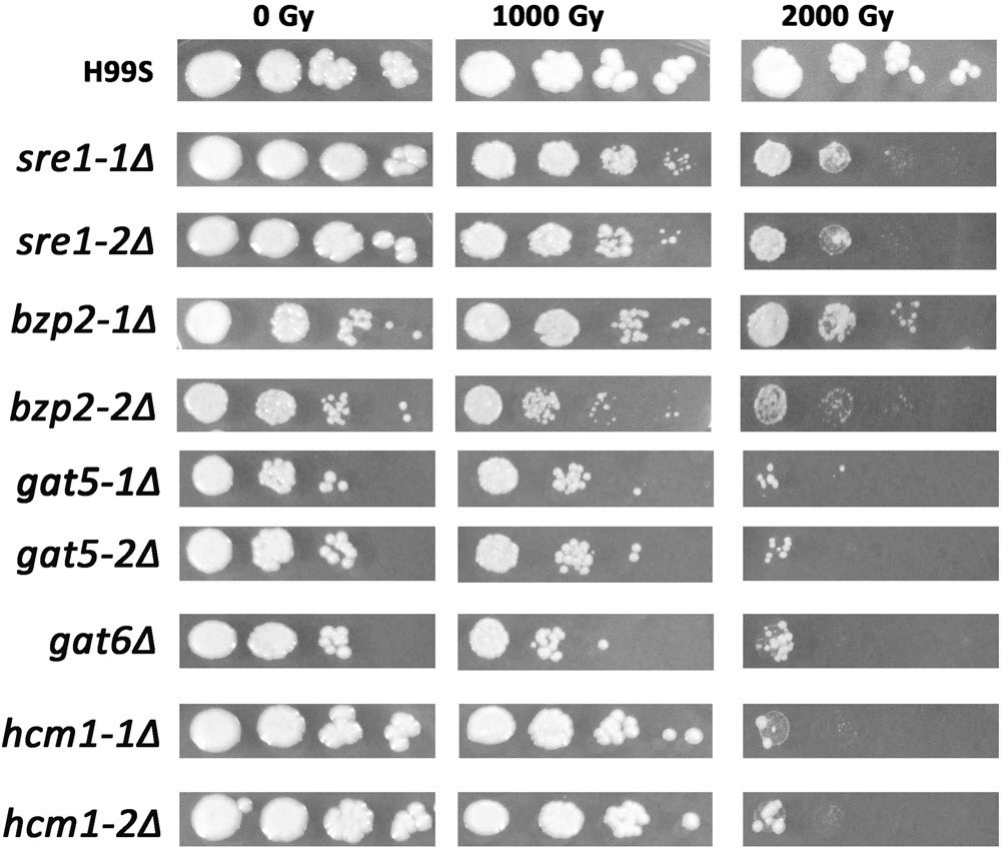
Identifying TF mutants that have different radiation sensitivity compared to the wild type *C*. *neoformans* H99S. Cells cultured overnight in liquid YPD medium were 10-fold serially diluted (10^2^ to 10^5^) in PBS and 3 µL of diluted solution was spotted onto the YPD plates. Cells were then exposed to the indicated doses of γ-radiation and further incubated at 30 °C for 5 days. Image was from one representative experiment of three independent experiments.

**Figure 2:**
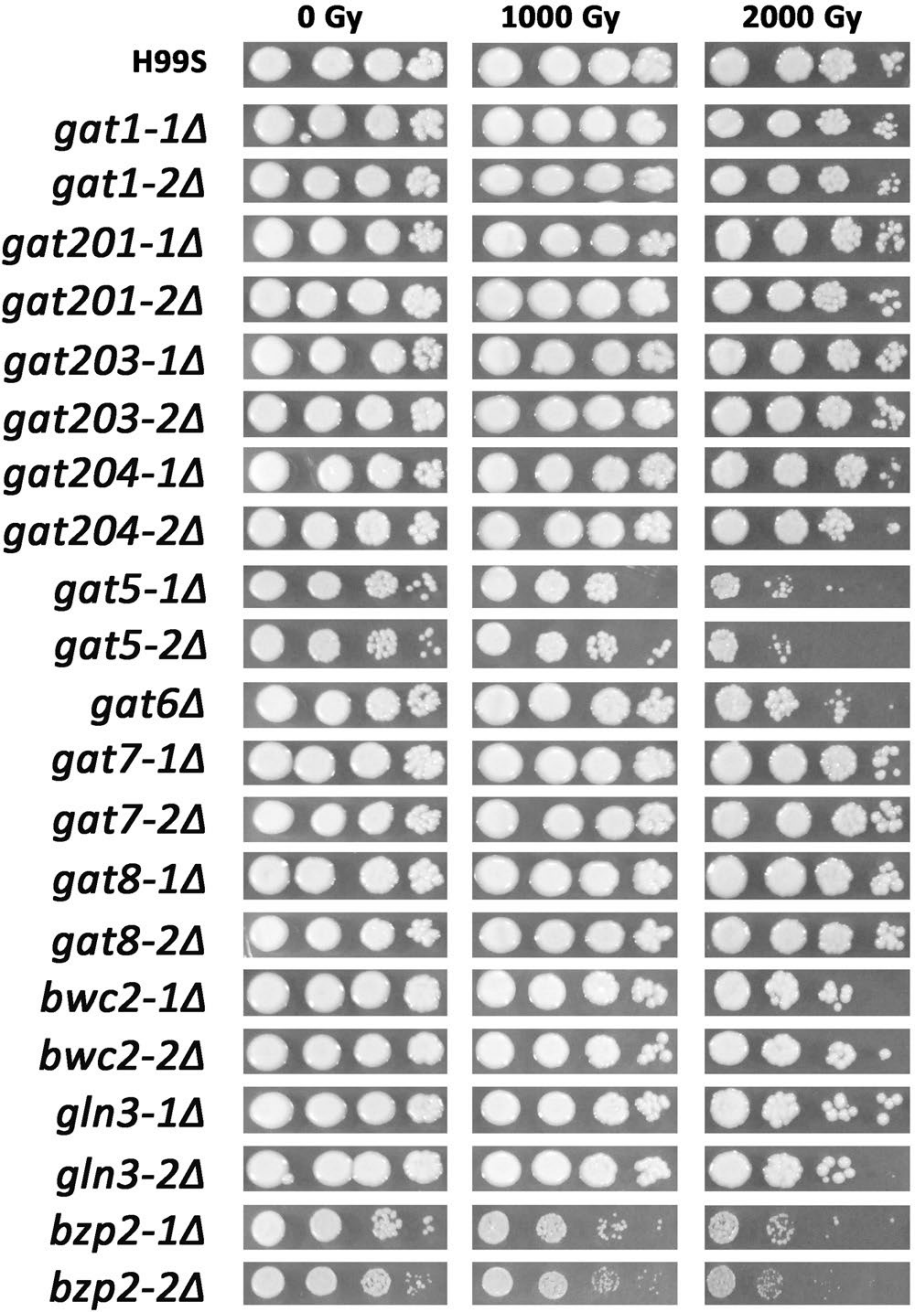
Radiation sensitivity of GATA TF mutants. GATA TF mutants and H99S cells cultured overnight in liquid YPD medium were 10-fold serially diluted (10^2^ to 10^5^) in PBS and 3 µL of diluted solution was spotted onto the YPD plates. Cells were then exposed to the indicated doses of γ-radiation and further incubated at 30 °C for 5 days. Image was from one representative experiment of three independent experiments.

We screened the mutant TF library to identify TFs that were important for radiation sensitivity. Cells were serially diluted and plotted on YPD agar and then exposed to 1,000 and 2,000 Gy γ-radiation. Among the 155 TFs, 5 TF mutants were shown to be more sensitive to IR than the wild type control H99S cells (Fig. 1). These included *sre1Δ*, *bzp2Δ*, *gat5Δ*, *gat6Δ* and *hcm1Δ*. *SRE1* is a TF important for sterol biosynthesis. In agreement with our findings here, it has been reported that *SRE1* mutant exhibited severe growth defects when exposed to γ-radiation^5^. *HCM1*, a Forkhead TF, has been reported to regulate chromosome segregation during S-phase^13^, which may explain why *hcm1Δ* mutant was sensitive to radiation. The three remaining TFs, *BZP2*, *GAT5* and *GAT6* all belong to the GATA TFs. To further confirm and also identify other potential GATA TFs important for radiation sensitivity, all the known GATA TF mutants in the mutant TF library were exposed to γ-radiation as done previously and their survival was monitored. Note that the collection of GATA factor mutants in Jung’s mutant TF library is not complete. For example, *CIR1* is a known GATA factor but it is not available in Jung’s TF library^11^. As shown in Fig. 2, only *GAT5*, *GAT6* and *BZP2* mutants were sensitive to radiation among all the tested GATA TFs as shown in Fig. 1.

### GATA TF mutants had different sensitivity to rapamycin, fluconazole or hydroxyurea treatment

**Figure 3:**
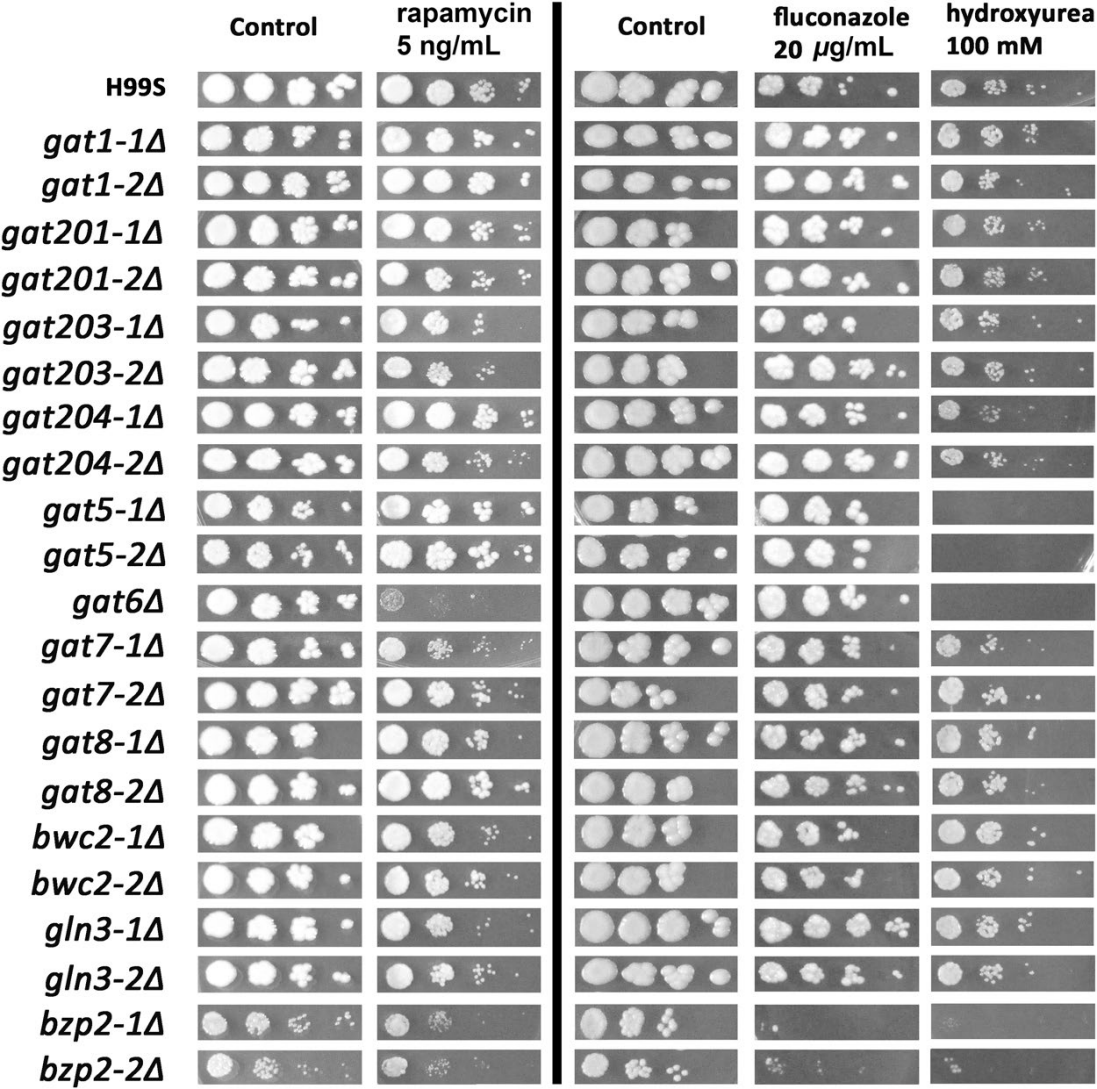
The responses of GATA TF mutants against rapamycin, fluconazole, or hydroxyurea treatment. GATA TF mutants and H99S cells were cultured overnight in liquid YPD medium were 10-fold serially diluted (10^2^ to 10^5^) in PBS and 3 µL of diluted solution was spotted onto the YPD plates containing the indicated concentration of agents. Image was from one representative experiment of three independent experiments. Note that the rapamycin experiment was done at a different time with fluconazole and hydroxyurea experiment as shown divided by the black line.

To test whether the GATA TFs are important for radiation only or for other stress responses too, we exposed the GATA TF mutants to rapamycin, fluconazole or hydroxyurea treatment. Rapamycin treatment mimics nitrogen starvation through inhibiting the mammalian targets of rapamycin (mTOR). Fluconazole is the most widely used antifungal drug. And hydroxyurea is a DNA damaging agent. As shown in Fig. 3, *gat6Δ* mutant exhibited poorest survival after rapamycin treatment; *bzp2Δ* mutant exhibited poorest survival after fluconazole treatment; similar to radiation exposure, *gat5Δ*, *gat6Δ* and *bzp2Δ* mutants were the most sensitive mutants to hydroxyurea treatment. The data suggest that there were specific GATA TFs important for different stressors.

### Characterizing the GATA transcriptional circuits after radiation exposure

**Figure 4:**
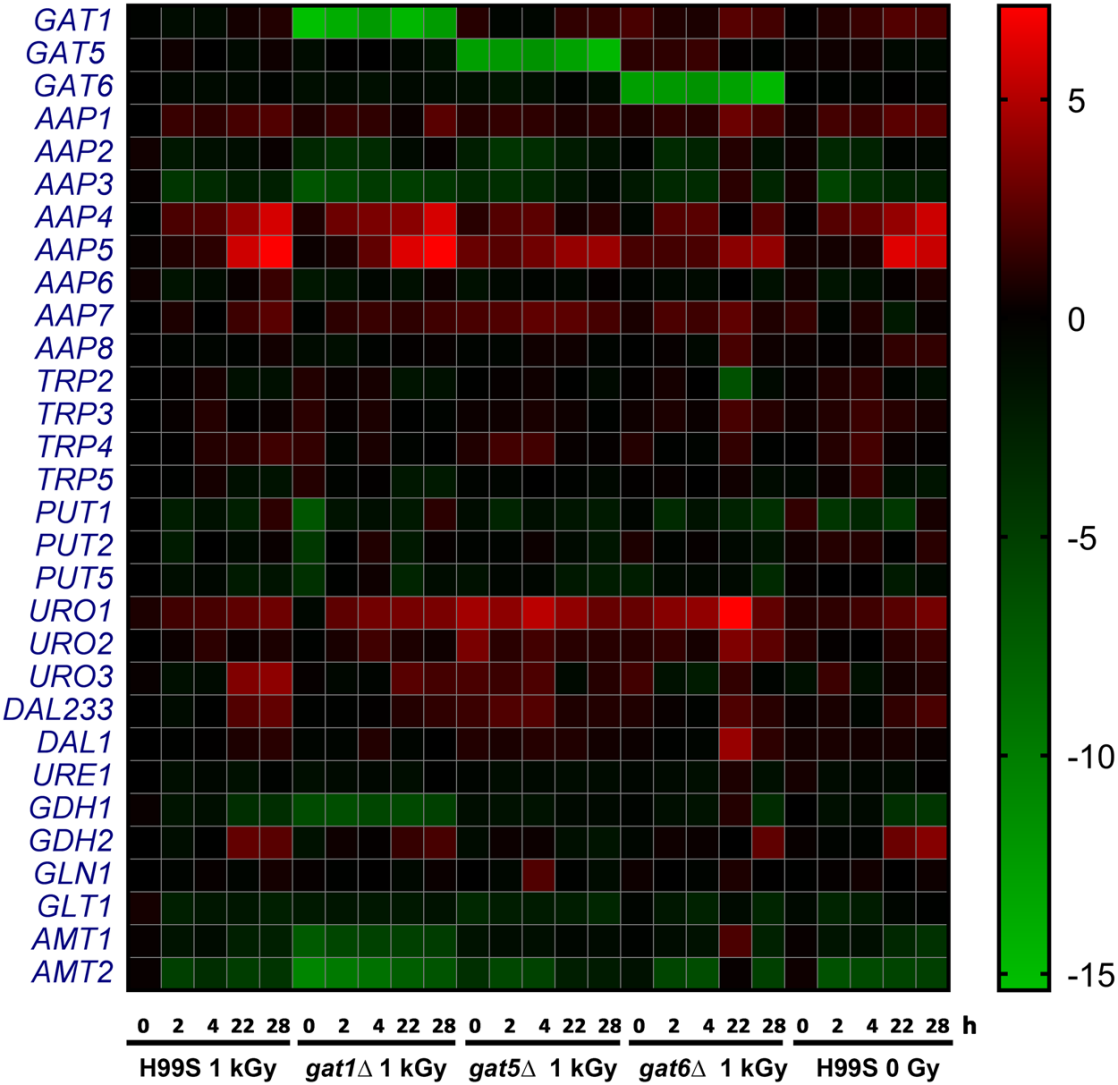
Heat map diagram of differential gene expression in wild type H99S and *gat1Δ*, *gat5Δ*, and *gat6Δ* mutants after radiation exposure and also H99S without radiation exposure. The relative gene expression level was log2 transformed. The key for the heatmap coloring is included on the right. Each square represents the average of at least two biological repeats.

**Figure 5:**
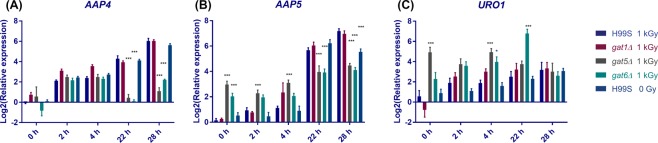
Differential gene expression of *AAP4*, *AAP5* and *URO1* in H99S and GATA mutants after radiation exposure and also H99S without radiation exposure. Gene expression was determined using qRT-PCR and normalized to actin. Gene expression levels were log2 transformed. Each group was compared to the H99S 1 kGy group at each time point. Each bar represents mean ± SEM of 4 biological replicates. (*P < 0.05, **P < 0.01, ***P < 0.001).

GATA TFs regulate a wide range of downstream genes involved in NCR; NCR is the physiological process that cells can preferentially utilize good nitrogen sources (such as glutamine, ammonium etc) instead of poor nitrogen sources (such as urea, proline, allantoin, glutamate, arginine, etc)^14–16^. In *C*. *neoformans*, GATA TF downstream targets include amino acid permeases *AAP1-8*^17^; uric acid catabolic enzyme-encoding genes *URO1-3*, *DAL1*, *DAL233*, and *URE1*^18^; proline utilization pathway genes *PUT1*, *2* & *5*^19^; ammonium assimilation enzyme-encoding genes *GDH1-2*, *GLN1*, *GLT1*, ammonium permeases *AMT1-2*^20^; tryptophan biosynthetic pathway genes *TRP2-5*^17^. To identify potential downstream NCR genes regulated by *GAT5*, *GAT6* and *BZP2* after radiation exposure, H99S and *gat1Δ*, *gat5Δ*, *gat6Δ* TF mutants were cultured and then exposed to 1 kGy γ-radiation. We included *gat1Δ* mutant because it was the most widely studied GATA TF in *C*. *neoformans*, even though it was not radiation sensitive. We also included the H99S un-irradiated samples to study the temporal effect on NCR gene expression. Cells were harvested at different time points after radiation. Gene expression levels were assayed using real time PCR. Both *bzp2Δ* mutants grew poorly in YPD broth, therefore, we didn’t study the gene expression in *bzp2Δ* mutants. All the gene expression levels were plotted in the heatmap shown in Fig. 4. There were no expression of *GAT1*, *GAT5* and *GAT6* in the *gat1Δ*, *gat5Δ* and *gat6Δ* mutant respectively, suggesting the complete deletion of *GAT1*, *GAT5* and *GAT6* genes in these mutants. Furthermore, there was no significant changes of *GAT5* or *GAT6* expression levels at different time points after radiation. This was in agreement with literature suggesting that GATA TFs such as *Gln3* and *Gat1* in *Saccharomyces cerevisiae* translocate to nucleus after dephosphorylation thus regulating NCR gene expression but not by changing the gene levels of GATA TF themselves^14^. In the *gat1Δ* mutant, *GDH1*, *AMT1* and *AMT2* expression was very low which is in agreement with a previous report^20^. Among the potential GATA downstream genes, *AAP4* and *AAP5* were highly elevated in the H99S and *gat1Δ* mutant at 22 and 28 h after radiation exposure. However, the levels of *AAP4* and *AAP5* were much lower in the *gat5Δ* and *gat6Δ* mutants at those time points. *URO1* level in the *gat6Δ* mutant was very high at 22 h post radiation. To better characterize the gene expression changes after radiation in the H99S and mutants, we focused on studying the expression of these three genes as shown in Fig. 5. There were similar levels of expression of *AAP4*, *AAP5* and *URO1* in H99S cells with or without radiation and *gat1Δ* mutant at each time point. For *AAP4* gene (Fig. 5A), there were no significant differences between each group at 0, 2, and 4 h after radiation. However, at 22 h and 28 h after radiation the expression levels of *AAP4* were significantly lower in the *gat5Δ* and *gat6Δ* mutants compared to the H99S irradiated group (at 22 h, the relative expression for *AAP4* was 15.51 ± 1.44, 1.46 ± 0.34, and 1.09 ± 0.10 respectively in H99S, *gat5Δ* and *gat6Δ* mutants. At 28 h, the relative expression for *AAP4* was 66.09 ± 5.40, 2.31 ± 0.53, and 4.63 ± 0.22 respectively in H99S, *gat5Δ* and *gat6Δ* mutants). For *AAP5* gene (Fig. 5B), there were small but significantly increased expressions at early time points in *gat5Δ* mutant at 0 h, 2 h and 4 h (relative expressions were 8.16 ± 1.21, 5.08 ± 0.92, and 8.88 ± 1.50 in the *gat5Δ* mutant vs 1.12 ± 0.12, 1.97 ± 0.26, and 1.21 ± 0.23 in the H99S 1 kGy at 0 h, 2 h, and 4 h respectively), and in *gat6Δ* mutant at 0 h compared to the H99S 1 kGy samples (relative expressions were 4.26 ± 0.71 vs 1.12 ± 0.12); at later time points, there were significantly decreased levels of *AAP5* in the *gat5Δ* and *gat6Δ* mutants (The relative expression at 22 h was 52.43 ± 7.78, 18.70 ± 7.20, and 15.80 ± 2.69 respectively in H99S, *gat5Δ* and *gat6Δ* mutants; the relative expression at 28 h was148.28 ± 18.92, 22.62 ± 2.60, and 17.75 ± 2.14 respectively in H99S, *gat5Δ* and *gat6Δ* mutants). The *URO1* gene had significantly higher level at 0 h post radiation in the *gat5Δ* mutant compared to the H99S 1kGy (36.12 ± 12.29 in *gat5Δ* vs 1.96 ± 0.96 in H99S); and it also had significantly higher level at 4 h post radiation in the *gat5Δ* and *gat6Δ* mutants (3.93 ± 0.88, 33.62 ± 8.66, and 19.34 ± 8.12 in H99S, *gat5Δ* and *gat6Δ* mutants respectively). In Fig. 5C, *URO1* gene was significantly higher in the *gat6Δ* mutant at 22 h post radiation compared to the H99S 1kGy group (7.14 ± 3.14 vs 124.30 ± 33.32 in H99S and *gat6Δ* mutant). Radiation alone did not change the levels of *AAP4*, *AAP5* and *URO1* at multiple time points as shown by the insignificant difference between H99S 1 kGy and 0 Gy samples, implying that radiation had no direct effect on the levels of these genes. The data suggest that *GAT5* and *GAT6* specifically regulate *AAP4*, *AAP5* and *URO1* genes with different dynamics. The *GAT5* and *GAT6* up-regulate *AAP4* gene expression at later time points; while *GAT5* and *GAT6* down-regulate *AAP5* gene expression at early time points, and then up-regulate *AAP5* expression at later time points. *GAT5* inhibits *URO1* expression at early time points and *GAT6* inhibits URO1 expression at later time points.

Fungal GATA TFs recognize an HGATAR (5′-(T/A/C) GATA(A/G)-3′) motif upstream of their target genes^21^. We analyzed 2 kb DNA sequences upstream of *AAP4* and *AAP5* genes, and identified 4 GATA binding sites for *AAP4* gene and 8 GATA binding sites for *AAP5* gene as shown in the Supplementary file.

### Phylogenetic analysis of the GATA factors in *C*. *neoformans*

**Figure 6:**
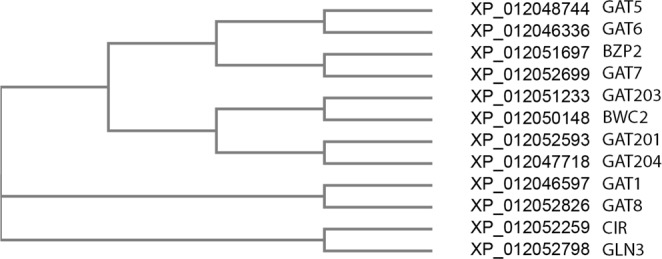
Phylogenetic tree of GATA TFs in *C*. *neoformans*. The protein sequences of GATA factors were used for phylogenetic tree analysis. Each GATA factor’s NCBI reference number is listed before the GATA TF names.

To obtain information about the evolutionary relationships of the GATA factors in *C*. *neoformans*, phylogenetic analysis were performed based on the protein sequence alignments of all transcribed proteins of the GATA family members (Fig. 6). Compared to other GATA factors, GAT5 and GAT6 were mostly closely related to each other. BZP2 and GAT7 were the next closely related GATA factors to GAT5/6.

## Discussion

In the current study, we identified TFs and their downstream genes that play important roles in radiation resistance in *C*. *neoformans*, which is a highly radiation-resistant fungus found in highly radioactive environment. Radiation-resistant microbes utilize many different strategies to counteract the damaging effects of ionizing radiation, such as high-levels of H-Mn2^+^ complexes to protect proteasome in *D*. *radiodurans*^1,22^ and efficient repair of DNA damages in cyanobacterium *Chroococcidiopsis*^23^ and *D*. *radiodurans*^24^. The molecular mechanisms of *C*. *neoformans* radiation-resistance are still not well understood. Transcription factors regulate coordinated gene expressions in response to various physiological or environmental signals. In our current study, we found that specific GATA TFs play important roles in *C*. *neoformans* after radiation exposure. GATA TFs are a class of highly conversed TFs present in fungi, metazoans and plants^16^. The fungal GATA TFs regulate many functions such as nitrogen metabolism, light induction, siderophore biosynthesis and mating-type switching^16^. There were few studies of GATA TFs in *C*. *neoformans*. Our data showed that even though they belong to the same TF family, each GATA TF may have specific functions. *GAT1* is the most widely studied GATA TF in *C*. *neoformans*, which regulates nitrogen uptake and controls the transcription of genes involved in NCR, ergosterol biosynthesis, iron uptake, cell wall organization and capsule biosynthesis^25^. However, *GAT1* mutant had the same sensitivity to rapamycin treatment as the wild type^25^, which is further confirmed in our current study. We showed that *GAT6* mutant was most sensitive to rapamycin treatment in the GATA family. Therefore, the TOR signaling pathway, which regulates cell proliferation and is the target of inhibition by rapamycin, may act through *GAT6* in *C*. *neoformans*. *GAT5*, *GAT6*, and *BZP2* mutants were all sensitive to IR and hydroxyurea, suggesting they are important for DNA damage responses. *BZP2* is the only GATA TF that is sensitive to fluconazole treatment. Our data suggest that specific GATA TF or their combination is important for different stressors. Furthermore, we showed that among the 28 downstream genes of GATA TFs we studied, only *AAP4*, *AAP5* and *URO1* were significantly changed in the *gat5Δ* and *gat6Δ* mutants after radiation (Fig. 5).

Studies in *D*. *radiodurans* radiation has suggested that IR resistance is predominantly a metabolic phenomenon, because *D*. *radiodurans* can grow on rich medium in the presence of continuous radiation (60 Gy/h) without lethality while *D*. *radiodurans* loses viability coupled with severe DNA degradation if grown in minimal medium and exposed to 60 Gy/h chronic IR, unless high concentrations of amino acids are provided^22,26,27^. Interestingly, it was shown in the 1960s that radiation inhibits amino acid uptakes in *Escherichia coli*^28^. In agreement with literature, our findings suggest that certain micronutrients are important for radiation resistance. We hypothesis that in response to IR, *GAT5* and *GAT6* in *C*. *neoformans* are required for the expression of amino acid permeases such as *AAP4* and *AAP5*, thus transporting more specific micronutrients to the cells, and increasing cell survival following radiation. The data suggest that supplementing specific micronutrients may increase cell survival following radiation as shown in *D*. *radiodurans*. Not only in microbes, it has been shown that micronutrients such as amino acid mixture mitigates radiation-induced gastrointestinal toxicity in mice^29,30^ and oral supplementation with amino acid preparation helps hepatocellular carcinoma patients undergoing radiotherapy^31^. It would be interesting to check whether GATA TFs and AAPs in other organisms are also important for radiation sensitivity. If that is the case, then identifying the specific substrates for the AAPs may provide better radio-protection reagents for cells or even animals.

In addition to their importance in radiation, *GAT5*, *GAT6* and *BZP2* were shown to be important for *C*. *neoformans* virulence^11^. Interestingly, *GAT5*, *GAT6* and *BZP2* had no effect on virulence-factor (capsule, melanin, and urease) production as shown in the phenotype database at tf.cryptococcus.org^11^. *C*. *neoformans* has to handle the various stresses found at infection sites such as nutrient limitation, high temperature, and other innate factors for successful proliferation, dissemination, and colonization^32,33^. Therefore, even though these three GATA factors are not required for virulence factor production, they may up-regulate downstream NCR genes, such as *AAP4* and *AAP5*, thus enabling cells to utilize poor nitrogen source for better survival. In other words, *GAT5*, *GAT6* and *BZP2* are important for virulence not by regulating the virulence factor production, but by regulating downstream NCR genes for better survival. In agreement with this hypothesis, it has been shown that *AAP4* and *AAP5* were essential for virulence in *C*. *neoformans* against high temperature and oxidative stress by mediating amino acid uptake during these stress conditions^34^.

The *AAP4* and *AAP5* gene expression showed a similar level of time-dependent increase in both the un-irradiated and irradiated H99S samples as shown in Fig. 5, suggesting that nutrition is a major regulator of *AAP4* and *AAP5* expression. We hypothesize that as the nutrients are being depleted in culture with time, *AAP4* and *AAP5* are being transcribed so that unfavorable amino acids can be transported into cells for better survival. Radiation will not directly regulate the expression of *AAP4* and *AAP5* genes. However, in irradiated cells, amino acids transported by permeases AAP4 and AAP5 are not only needed for starvation-related survival, but also needed for the repair of radiation-induced macromolecular damages. Therefore, cell survival after radiation is being determined by the availability of specific amino acids transported by AAP4 and AAP5.

The phylogenetic analysis showed the close relationship between GAT5 and GAT6 (Fig. 6). Interestingly, previous work also showed that AAP4 and AAP5 are mostly closely related within the 8 amino acid permeases^17^. So potentially a subfamily of GATA factors exists to respond to different stressors. For example, the GAT5/6-AAP4/5 subfamily is most important in response to ionizing radiation; while the GAT1-GDH1/AMT1/2 subfamily may be more important in response to nitrogen starvation.

**Figure 7:**
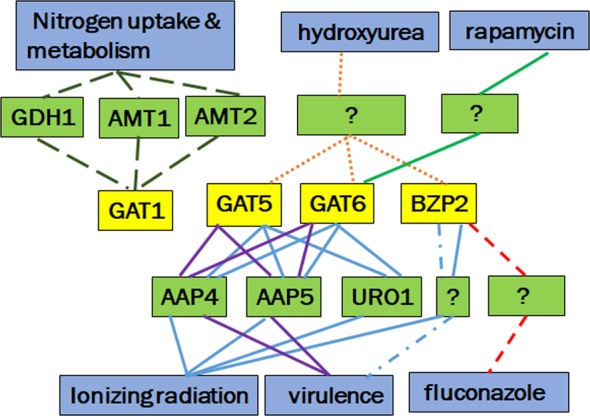
Summary of the GATA TF circuits important for different stressors in *C*. *neoformans*. The yellow boxes are GATA factors, green boxes are downstream genes of GATA factors, and the blue boxes are related biological processes. The subfamily of GATA factors important for one biological process is linked by the lines with same color/pattern.

We summarized the findings of GATA TF circuits in response to different stressors in Fig. 7. Specific GATA TFs are important for *C*. *neoformans* in response to different stressors. *GAT1* is responsible for nitrogen uptake and metabolism but not important for rapamycin treatment as reported earlier^25^, mixed results for virulence^11,20^, also not important for radiation resistance. *GDH1*, *AMT1* and *AMT2* are important down-stream genes of *GAT1*. *GAT5*, *GAT6* and *BZP2* are important GATA TFs for radiation resistance and virulence. *GAT5* and *GAT6* regulate *AAP4*, *AAP5* and *URO1* genes in response to radiation. *GAT5* and *GAT6* regulate *AAP4* and *AAP5* in virulence^11,34^. *GAT5*, *GAT6* and *BZP2* are also important GATA TFs for another DNA damaging agent hydroxyurea treatment. *GAT6* is important for rapamycin treatment while *BZP2* is important for fluconazole treatment.

In conclusion, we identified important TFs for radiation sensitivity in *C*. *neoformans*. Not only important for radiation response, specific GATA TFs are also important in response to other stressors. Particularly, *GAT5* and *GAT6* may control radiation sensitivity by regulating the expression of downstream genes such as *AAP4*, *AAP5* and *URO1*.

## Supplementary information

AAP4 and AAP5 binding sites

## Data Availability

All data generated or analyzed during this study are included in this published article.
