# Supplementary material for: GATA-type transcription factors play a vital role in radiation sensitivity of Cryptococcus neoformans by regulating the gene expression of specific amino acid permeases
Source: Sci Rep. 2019 Apr 23;9:6385. doi: 10.1038/s41598-019-42778-7 (PMC6478845; doi:10.1038/s41598-019-42778-7)
Supplement: Supplementary file 1 — AAP4 and AAP5 binding sites [file 41598_2019_42778_MOESM1_ESM.pdf]

Title: GATA-type transcription factors play a vital role in radiation sensitivity of *Cryptococcus neoformans* by regulating the gene expression of specific amino acid permeases

Authors: Wanchang Cui, XiangHong Li, Lisa Hull, Mang Xiao.

The GATA binding sites of *AAP4* and *AAP5* genes in *Cryptococcus neoformans*:

AAP4 gene (Genbank access number XM_012191441) spans H99 chromosome 1 (Genbank access number CP003820) from 1533591- 1536730. There are 4 GATA binding sites within 2 kb DNA sequence upstream of *AAP4* gene as highlighted.

>gi|405117359|gb|CP003820.1|:1531591-1533591 Cryptococcus neoformans var. grubii H99 chromosome 1, complete sequence
TTTCTATGCCCATCTACAGCACCTTGTTGTTCAGAATACGGGTACTGTGGAACAGATGGCTACTGTCTGG
GAGGTTGTGAGCCACTGTGTAAGCCAACTATAGCTATCTATGGGTGGTGATGTAGCTGACAGGATGCAGA
CTCCCACGAAGTCACTTCATGCCGGCCAGATCCTATCTGTACTTCCTTCAAGACAGACTTCACTGACCTT
AGCAGGGTTCAACAGAATGTATCTCTGTACGACGGTAATGCCACAGCGTACGATTGGGTTGGTGAGTACG
ATGGAAAATGTCTGCTCCAGGCTGTGCTAAAACCTGTCAGTTAACACGGGATCCCTGATCGCTTCATCCG
ATAATGATGGAGCTCGTCTCATCCTAACCCAGAATAACACTGGTTCCAAAATATCTAGTACCCGTTACCT
CCATTACGGAACTATCGACTTTACTTTGCAGACGTCAAAATGGGCCGGTGTGGTGACAGCAGCAATCACA
ATGTCCGACGTCAAGGATGAAATAGATTGGGAATGGCCTGGGGCTAATACCGATACGGTGCAGACAAACT
ATTGGTTCTTGGGTATTGCAAACTGTAAGCAGCAAGTGCCGAGATAATTAGCTGATAATAGACTCGGCGA
CGGAGGGTGCTTCCAGCTCGGTGTCTTCTGATACGGCTGCCCACTTTCACACCTACACTGTAAATATTAA
TGGAGTGACGTAGCGTTCGACTGACCCAATGACAGATTGACTGGCAAGAGAGTTACATCAGTTGGAGTAT
TGACGGGTTACTTGTTCGTACAGTCAGGAAGACAGATACCATTTCTGAAGACGGGACACAGTAGTAAGCA
AGCCTCTCACTCATCTCATAACATAGCAGTACAGAACAAAGGCTTGTAGATCTGACGTTTCTGCAGTAAA
TTCCCCTCCACTCCATCTCGTGTTCAAATCTCCATTTGGCCCGCTGGCACAGACGGCAATGCTCAGGGCA
CTATCGATTGGGCGGGTGGCTACATCGATTGGACAGATAATGACTACCTGTCCAAGGGGTATTTCTGGAA
CACCATCAAGAGTGTCAATATCAGCTGCGCATATGACACTGCTTCTTCTGGGGCGACAGGCTGGGCTTAC
TCTGGAAATGATACATCGGGGATACCGGTGAGTCCAACAATCTCATTTTCTTTTCCCTATTACTCCCTCT
CGTTCAACCGAGGCGACGACGAACAAGGAAAGTTAACGAGAGATGTTGACGTTTTATCATTTATCTCAGA
TTGTGACCACTACCAATGCGTCAACTCTGATCTCCGCCGGAAGGAAGAGAATTGTAGGAGTCGAGATCAA
TAGTCGAGGGATGGGTGGAGTGGGTCTGCTTATCATCAGTGGCTTTACATTGCTCGGAGGCGTAGCTCTC
ATCTAATCTTCTATGTCCGATGGTAACTGTACAGTGTTGAAAATTACGTATCTTTGCCATCGGTCGATGG
CGTTTGATTCAAAATAGATATGATATGAGATTTACCAAATTAGATCAATATGGGCCATTCATACTTATTA
ACCTGCTGGACGCTCGGAAAAAAGCATACGCATATTATGAATGACGGAGAAGAGGAGGCGAGCAATATAC
TTATATAAGTATAGTATGCCGTACATAGTGCATCAATCACCTTTACAGCCTCCCCACTCTTTCCCCCCAT
TCATAAAATGGTGAGCACACCATGTAAAATCATTAATTTCATTGTTTAGTTCGATAGTGATTTTGCTCTA
AATGTTCAATGATCTAATAGCAGTAATGAGTGCACGTAATATCTCAGTCTGATATCAGGGTTCAGCAGCT
GTGTAGCTGGCAGCCAAATGGGGCCAACAAGTTCCGCATGGGTAATGGTGACGGGTGACGGTGAATGAGC
GAAGCGGCATCATTTGTTCTCATTCTAGTCAGCGAAAATGACAAATGTCAGAAGCTAGAAGATGTATAAA
AAATACAAGTCACATTACTGAAAACTCACGAAGCTCCATTG

AAP5 gene (Genbank access number XM_012191669) spans H99 chromosome 1 (Genbank access number CP003820) from 1424618-1422185 (Note the **minus** strand). There are 8 GATA binding sites within 2 kb DNA sequence upstream of *AAP5* gene as highlighted.

>gi|405117359|gb|CP003820.1|:1420185-1422185 Cryptococcus neoformans var. grubii H99 chromosome 1, complete sequence
AGATAACAGTTTTCATGACTGGCACAAAAGCAGACTCAACCGCCTACTTCATTCTCAGCACGGTTCCAGA
ATGTCTGTAATGAGCTCACAGAAATTTCAGTAACTGCTGGCCAAGCACGTAGCATATCTTGAAATTCACC
AAAGAAATGTTCTCTGTAGATATGCCGCGGGCAGACACAAATAGTATCAATATCAGACCCAGGGCCGTGA
ACACCGAGACTATGTACTAAGTTAGCCATTGAACTCGATATATCGAATTTACCCACCGATAAGAACCTGA
AGTATAGATTCGTCCACCAGCCTCTGAAGCTATTTTTTCTGACATGCCGAGTTTTATTGATACATCATGA
ACAAATTTAGCAACCAGCTGGGCGATATTTGAAAGCAATCGTTCTCTGCATCCAAATAAGCCTTCATCTT
CGCTTTTCCAGCTCAACGCAACATACCGAACCTTCCTTTCTTGATCAGATTCGAATTGATTCAGGGCCAC
AAGGTCCGCCATCAAAGCTTCACTAGACTTGATATCATTGGGCTTTGGAGGATCAGTCGTAATGGGAGGT
GTAACACCCAAGAACTTGACAGGCGGGTTACTGTTGTTCACTATCGTTAGTCTACACCAGGAATTGCCGC
TTCCAGACGGAATGACCAGCAATAGCACGCACGAAGTCATTGGTGTTTTCTGACAGATAGGAAGAACAAC
TCGGGGCTTGGAGTAAGACAGTCGAAATGAAGTTCGGTAATCGCAAGAGCCCAGTCGTGAAATAATTTGG
TATTCGGTGCTCAAATTCTTTGCAGCTACGTCAATTAGGTGCTGTACATCAATTGCTGATTCTTGATTAT
GAAGAAATGTAGAGATCAAGAAGCAACTAGAGATACGATACGTAAAGCACAAAGAACACCTTAGACGCTG
TTGTATCCTCGGTGGCAGTCGGAGTTATCCGCTGTGGCACTAATAATATGCAGCCGTGCTGGAGTCTCCT
CACCAGAGGAAAGGAGGCAAAACAATAGTTGTTCGTACCTTTTTAAGCATGATCCATTATACAAATTTGA
CGCCACTTATGTGTACGTGAGGGCCCGTTGTCCCTAGCGGACATCCACAGAGCTTTTTCTGCGCTCGTCC
TCTCTCTCACCCTCCACGAAGACTGACGGGGCCCGCCACCCGGCAACTAATAATAAAAGAAGCGCTCACC
GTACGCGCAGTAGAATTATCGTCACCCTTGTGCTCCCTTTTACCTGTAAGCCAAGCGGATCGCCCTTTGA
GCTAATTCTGGAGATTTATGATAAGCTCCTAGGAAAGAGGAAATGAGCAACGCGTTGATGCGAGGAGTAA
AAGCCGATGAGGACGAAGAAGAGAGGTGCGCCGAGCCGGATGTGTTGTTGTGAGGTTTGGGGAAGGTGCG
AATTCCCCGACTAAACAACATTCATCACTGCGGCCCAGCGGCAAGCTTCCATCGTTTACAATCCATTAAT
ACGTGACTATATATTTCTAAAACATAAGAGTTACTTTTACTAAAAACTGTTCTACTCCCAACCTAATCTA
ATCTTCCCTATCTTCATTCTTCGTCCATCTCTTCATTTCTTCCTGTTTTCTCTTACAATCCGACTTCCGT
TTGAGACGACCAGGAATTTAAATCAATCCCTCCCCCCACGGAAACAATAAGAAGCGGCGCTGGCCATGTG
AGCCCTGCTGCCCTCTTCTGCAGCGACCTGAGACACCAGCGTAGGACAAGGTCGCAGGATGACTTCCCCG
ATTCTCGCTTGTTGGCCGTAGTGTGGAGCGGGAGTTGTGCTGATGGACAGAGTTGTAAGACATTAGGTCA
ATGGACTTGTCGTAAGTGCGATGGTTCCACTGCGCAATATCATAATTGGCATCTCACTAGGCCCATTACT
AGGATGCATAGGTATATCTCATTCTGTGGACGCACGCCCATTTTGGGCCTCCTCCGATTAAGATACCTCA
TTAACCGACGCAGGCTCCTGAGTTTGGGTGCTTCGCGCTGC
